# Molecular and process design for rotavirus-like particle production in *Saccharomyces cerevisiae*

**DOI:** 10.1186/1475-2859-10-33

**Published:** 2011-05-14

**Authors:** William A Rodríguez-Limas, Keith EJ Tyo, Jens Nielsen, Octavio T Ramírez, Laura A Palomares

**Affiliations:** 1 Departamento de Medicina Molecular y Bioprocesos. Instituto de Biotecnología. Universidad Nacional Autónoma de México. Apdo. Postal. 510-3. Cuernavaca, Morelos, CP. 62250, México; 2 Systems Biology, Department of Chemical and Biological Engineering, Chalmers University of Technology, Kemivägen 10, Gothenburg, SE-41296, Sweden

## Abstract

**Background:**

Virus-like particles (VLP) have an increasing range of applications including vaccination, drug delivery, diagnostics, gene therapy and nanotechnology. These developments require large quantities of particles that need to be obtained in efficient and economic processes. Production of VLP in yeast is attractive, as it is a low-cost protein producer able to assemble viral structural proteins into VLP. However, to date only single-layered VLP with simple architecture have been produced in this system. In this work, the first steps required for the production of rotavirus-like particles (RLP) in *S. cerevisiae *were implemented and improved, in order to obtain the recombinant protein concentrations required for VLP assembly.

**Results:**

The genes of the rotavirus structural proteins VP2, VP6 and VP7 were cloned in four *Saccharomyces cerevisiae *strains using different plasmid and promoter combinations to express one or three proteins in the same cell. Performance of the best constructs was evaluated in batch and fed-batch cultures using a complete synthetic media supplemented with leucine, glutamate and succinate. The strain used had an important effect on recombinant protein concentration, while the type of plasmid, centromeric (YCp) or episomal (YEp), did not affect protein yields. Fed-batch culture of the PD.U-267 strain resulted in the highest concentration of rotavirus proteins. Volumetric and specific productivities increased 28.5- and 11-fold, respectively, in comparison with batch cultures. Expression of the three rotavirus proteins was confirmed by immunoblotting and RLP were detected using transmission electron microscopy.

**Conclusions:**

We present for the first time the use of yeast as a platform to express multilayered rotavirus-like particles. The present study shows that the combined use of molecular and bioprocess tools allowed the production of triple-layered rotavirus RLP. Production of VLP with complex architecture in yeasts could lead to the development of new vaccine candidates with reduced restrictions by regulatory agencies, using the successful experience with other yeast-based VLP vaccines commercialized worldwide.

## Background

Virus-like particles (VLP) are obtained when viral structural proteins are produced in heterologous expression systems in absence of viral genetic material. VLP assemble without the action of non-structural viral proteins and have the same architectural characteristics as native viral particles. The current market demand of VLP is primarily as products for vaccination [1], but other applications include diagnostics, biomedicine, material science and nanotechnology [2,3]. VLP have successfully protected humans and animals from viral diseases [4], inducing excellent humoral and cellular immune responses [5,6]. To date, a few VLP-based vaccines are currently used for human vaccination worldwide: hepatitis B and human papillomavirus vaccines are commercialized by several pharmaceutical companies. Other VLP-based vaccine candidates are in clinical trials or undergoing preclinical evaluation, such as influenza virus, parvovirus, Norwalk and various chimeric VLP [7,8]. VLP have been expressed in different heterologous systems, from bacteria and yeasts to the insect cell/baculovirus system (IC-BVS) and various mammalian cell lines. Ease of expression, ability to scale up and cost of production have made yeast the most popular VLP expression system [9]. However, to date yeasts have only been used to produce simple VLP, formed by a single protein layer.

*Saccharomyces cerevisiae, Hansenula polymorpha *and *Pichia pastoris *have been used to produce single-layered VLP of different viruses composed of a single nucleocapsid protein or a chimeric protein assembled in one layer [7,10]. The production of multilayered VLP in yeast imposes various challenges. First, several recombinant proteins need to be simultaneously produced in each cell in conditions adequate for their self-assembly into VLP. Second, the intracellular concentration of each structural protein should be high enough to promote VLP assembly. Third, the intracellular environment should be adequate for assembly.

In this work, rotavirus-like particles (RLP) were used as a model for multiple layer VLP production in yeast. Rotavirus is the major etiological agent of gastroenteritis in children and young animals [11,12]. Each year, rotavirus causes the death of approximately 610,000 children and 39% of hospitalizations for diarrhea worldwide [13]. Moreover, rotavirus is an important cause of economic losses in animal production, including death, loss of weight and treatment of affected animals [14,15]. RLP represent an interesting alternative to traditional vaccines that are now in the market, as they are efficient immunogens, cannot revert to infectious forms, do not need to be inactivated, handling of potentially pathogenic viruses is not needed, and new recombinant vaccines for new serotypes can be rapidly and easily produced [8,9].

Rotavirus-like particles have been frequently expressed in the insect cell-baculovirus expression system [8]. This system has proven to be productive and versatile. However, insect cell culture requires high cost media and baculovirus manipulation requires highly skilled personnel. In contrast, yeast expression systems are easy to scale-up and highly productive.

RLP production requires the simultaneous expression and assembly of three recombinant proteins, 120 molecules of VP2 (inner layer), 780 molecules of VP6 (middle layer) and 780 molecules of the glycoprotein VP7 (outer layer), into triple-layered particles (tlRLP) [16]. In this work, molecular strategies to simultaneously express the three recombinant proteins in *S. cerevisiae *and molecular and process strategies to increase their concentration in yeast cells were developed. To our knowledge, this is the first time that double or triple-layered VLP are produced in yeast cells.

## Methods

### Yeast strains and plasmids

**Figure 1:**
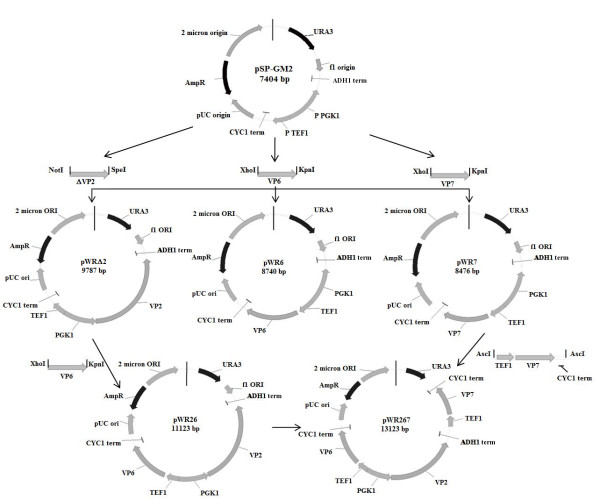
**Plasmids constructed in this work**. The ΔVP2 gene was cloned under the PGK1 promoter and VP6 and VP7 genes were cloned under individual TEF1 promoters. pWR26 was obtained by cloning the VP6 gene in plasmid pWRΔ2. The cassette TEF1-VP7-CYC1term was amplified by PCR from pWR7 and cloned into pWR26 to obtain pWR267.

Four *Saccharomyces cerevisiae *parental strains were used in this work: CEN.PK113-5D, W303-1a, PD 83B.1d and PJ69-4a. From these parental strains, 12 strains were constructed (Table 1). The rotavirus VP2 gene was obtained from the bovine rotavirus strain RF. To facilitate VP2 expression, the coding sequence for its first 92 amino acids, not necessary for VLP formation [17], was eliminated, and the ribosome binding site (RBS) sequence was optimized as previously described [18]. The new RBS sequence (TTCAAACAAA) was inserted by PCR before the initial ATG codon. This truncated VP2 is identified as ΔVP2 in Figure 1 and Table 1. The VP6 gene was obtained from the NCDV rotavirus strain and the VP7 gene from the MX002 strain [19]. All rotavirus genes used in this work have a bovine origin, and their compatibility was confirmed by their amino acid sequence homology to rotavirus NCDV proteins using BLAST (accession numbers: X14057, DQ870494, DQ870496, X65940, M12394, and FJ217205). The three genes were cloned in the pSP-GM2 plasmid, a variant of the pSP-G2 plasmid, recently developed by Partow et al. [20], for multiple gene expression. The cloning strategy is presented in Figure 1. To compare the expression levels in different plasmid types, the VP6 gene was also cloned in the centromeric plasmid (pRS7) under the PMA1 promoter. Yeast transformation was performed as described previously [21]. Table 1 lists the plasmids constructed and the strains used in this study.

**Table 1:** Yeasts and plasmids used in this study. Parental strains and their derivatives are shown

Strain	Genotype	Plasmid	Plasmid description	Reference
W303-1a	MATa ade2-1 can1-100 ura3-1 leu2-3,112 his3-11,15 trp1-1	-		
W.TLU-C		pRS7, pRS2, pSal4	P_PMA1_, CEN6/ARSH4, TRP1 P_PMA1_, 2 μ, LEU2, P_CUP1_, 2 μ, URA3	This study
W.T-6^d^		pRS7VP6	P_PMA1_VP6, CEN6/ARSH4, TRP1	This study
CEN.PK.113-5D	MATa SUC2 MAL2-8c ura3-52	-		Peter Kotter^a^
CEN.U-C		pSP-GM2	P_PGK1_, P_TEF1_, 2 μ, URA3	This study
CEN.U-6^d^		pWR6	P_PGK1_, P_TEF1_VP6, 2 μ, URA3	This study
CEN-U-267^e^		pWR267	P_PGK1_ΔVP2, P_TEF1_VP6, P_TEF1_VP7 2 μ, URA3	This study
PD 83B.1d^b^	MATa LEU ura31 TRP HIS ADE can1100 GAL SUC2	-		Stefan Hohmann^c^
PD.U-C		pSP-GM2	P_PGK1_, P_TEF1_, 2 μ, URA3	This study
PD.U-6^d^		pWR6	P_PGK1_, P_TEF1_VP6, 2 μ, URA3	This study
PD.U-267^e^		pWR267	P_PGK1_ΔVP2, P_TEF1_VP6, P_TEF1_VP7 2 μ, URA3	This study
PJ69-4a	MATa trp1-901, leu2-3,112, ura3-52, his 3-200, gal4D gal80D	-		
PJ.U-C		pSP-GM2	P_PGK1_, P_TEF1_, 2 μ, URA3	This study
PJ.T-6^d^		pRS7VP6	P_PMA1_VP6, CEN6/ARSH4, TRP1	This study
PJ.U-6^d^		pWR6	P_PGK1_, P_TEF1_VP6, 2 μ, URA3	This study
PJ.U-267^e^		pWR267	P_PGK1_ΔVP2, P_TEF1_VP6, P_TEF1_VP7 2 μ, URA3	This study

a. J. W. Goethe Universität, Germany

b. Strain with W303-1a background

c. Gothenburg University, Sweden

d. Contains the VP6 gene.

e. Contains the VP2, VP6 and VP7 genes.

### Culture media

Batch cultures were performed in a complete synthetic medium (CSM) without uracil. The medium contained glucose 20 g/L, yeast nitrogen base (Sigma-Aldrich Inc., St. Louis, MO, USA) 6.7 g/L and CSM -ura 0.77 g/L (Foremedium, Hunstanton, UK). Strains PJ.T-6 and W.T-6 were cultured in CSM without tryptophan. Media composition was improved using data from two two-level factorial designs of experiments in shake flask cultures, using VP6 and biomass concentration as response variables (data not shown). Based on the obtained results, culture medium was supplemented with leucine 1.8 mM, glutamate 20 mM and succinate 50 mM. pH was adjusted to 5.0 with KOH 2 N before autoclaving. Feeding medium for fed-batch cultures was 10 X CSM without supplements.

### Cultivation of recombinant yeast

All cultures were performed in triplicate in parallel 1L bioreactors (DasGip, Jülich, Germany) with a working volume of 0.7 L. pH was controlled at 5.0 with KOH 2N. The sparged air flow rate was 42 L/h (1 vvm). Carbon dioxide and oxygen concentrations in the outlet gas were measured with a DasGip GA4 gas analyzer. Cultures were inoculated to an initial OD_600 _of 0.01 with a liquid preculture.

The initial volume in fed-batch cultures was 0.4 L. Feeding followed an exponential rate and started when the carbon dioxide transfer rate value (CTR) decreased to 2.5 mM/h, which indicated glucose depletion. The feeding profile of the culture is expressed as follows:(1)

where:(2)

and *F *is the feeding flow rate; *b*, a feeding constant (0.19 h^-1^), x_0 _is the initial biomass concentration at the end of the batch phase; *Vo*, the initial volume (0.4 L); *t*, time; *Y_s/x_*, the substrate over biomass ratio (assumed to be 6.06 g glucose/g DCW for all strains); *C^f^_s_*, the substrate concentration in the feed (200 g glucose/L); and *C_s_*, the substrate concentration at time *t*.

### Analytical methods

Dry cell weight was measured by filtration through a 0.45 μm pore-size nitrocellulose filters (Sartorious, Göttingen, Germany) as previously described [22].

Glucose, glycerol, ethanol, pyruvate, succinate and acetate concentrations were measured in culture supernatants by HPLC (UltiMate^® ^3000 Standard LC system, Dionex, California, USA) using a HPX-87H Aminex column (Biorad, Richmond, CA, USA) at 50°C with 5 mM H_2_SO_4 _as mobile phase at 0.6 mL/min.

Recombinant proteins were extracted from the cell pellet using YeastBuster protein extraction buffer (EMD Biosciences, Inc, Darmstadt, Germany) following the manufacturer's instructions. VP6 was quantified by ELISA (ProSpecT, Oxoid, Cambridge, UK). VP2, VP6 and VP7 were detected by immunoblotting with monoclonal antibodies at different stages of purification. Briefly, 200 μL of each sample were filtered through a nitrocellulose membrane using a Bio-dot^® ^apparatus (Biorad, Richmond, CA, USA). Membranes were blocked with 5% non-fat dried milk in phosphate buffered solution (PBS), incubated for 1 h with monoclonal antibodies in PBS with 0.1% non-fat milk (3A8 for VP2, 255 for VP6 and IC3 for VP7, kindly provided by Drs. Arias and López, IBt- UNAM, Mexico). All monoclonal antibodies used bind conformational epitopes. Therefore, non-denaturing conditions are required for detecting rotavirus proteins with them. Membranes were washed three times with PBS with 0.1% non-fat milk, incubated for 1 h with a goat anti-mouse antibody conjugated with peroxidase (Jackson, Immunoresearch laboratories, West Grove, PA, USA), and washed three times. Peroxidase activity was detected by reaction with carbazole. Inactivated rotavirus strain SA-11 was used as positive control.

RLP were recovered as follows: Yeast intracellular extracts were ultracentrifuged over a 35% sucrose cushion at 112,700 × g for 2 h at 4 C in a SW-28 rotor and an Optima L-90K ultracentrifuge (Beckman Coulter, Fullerton, CA, USA). The pellet obtained was resuspended in TNC buffer (50 mM Tris-HCl pH 8.0, 0.15 M NaCl, 10 mM CaCl_2_). Cesium chloride was added to resuspended pellets to a concentration of 0.42 g/mL. Sample was centrifuged at 148,930 × g for 18 h at 4°C in a SW 55 Ti rotor (Beckman Coulter, Fullerton, CA, USA). Opalescent bands were isolated and observed by transmission electron microscopy (TEM). Samples were visualized by negative staining: Twenty five microliters of sample were fixed for 1 min in a 200 mesh grid coated with formvar-carbon (Structure probe Inc., West Chester, PA. USA). The grid was washed with ultrapure water and stained with 3% uranyl acetate (Structure probe Inc, West Chester, PA. USA), rinsed and left to dry. Samples were observed in a transmission electron microscope Jeol 1200EXII (Jeol, Peabody, MA. USA) operated at 80 KV.

Cushion sucrose pellets and CsCl gradient bands were also analyzed in sodium dodecyl sulfate (SDS)-12% polyacrylamide gels stained with Coomassie Brilliant Blue or blotted onto a PVDF membrane (Immobilon-P, Millipore, Billerica, MA, USA) in a wet chamber. Membranes were blocked with PBS-5% non-fat dry milk and incubated with a rabbit anti-rotavirus serum for 2 h at room temperature. Membranes were washed twice with PBS-0.1% non-fat dry milk and incubated with a peroxidase-conjugated goat anti-rabbit IgG antibody (Jackson, Immunoresearch laboratories, West Grove, PA, USA) for 1 h at room temperature. Membranes were washed three times with PBS-0.1% non-fat dry milk and peroxidase activity was detected by reaction with the chemiluminescence substrate Western Lightning Plus ECL (Perkin Elmer Inc, Waltman, MA USA). Gels and membranes were scanned and analyzed using a ChemiDoc system and the Image Lab 2.0 software (Biorad, Richmond, CA, USA).

## Results

### Molecular strategies for the simultaneous expression of three rotavirus genes

#### Plasmid and strain selection

tlRLP production requires the simultaneous presence in each cell of three rotavirus proteins, VP2, VP6 and VP7, which can be pursued by transforming cells with three plasmids, each containing one gene and complementing one auxotrophy, or by constructing a single plasmid containing the three genes. In order to assess the best of these two approaches, growth kinetics of two strains were determined, one containing a single centromeric plasmid expressing VP6 (W.T-6) and one containing three empty plasmids (W.TLU-C): one centromeric single copy plasmid (YCp) and two episomal high copy plasmids (YEp) (Table 1). Table 2 shows the maximum specific growth rate and biomass yield on glucose of both strains in batch culture. Three plasmids in the same cell decreased the maximum specific growth rate by 36%, compared with the strain with a single plasmid, but it did not significantly influence biomass yield on glucose. The W.TLU-C strain reached its maximum biomass concentration 12 h later than the W.T-6 strain. These results show that using three plasmids increased the metabolic burden in auxotrophic yeast, probably due to the need to complement three auxotrophies or to the burden of producing multiple copies of each of the three plasmids. A higher metabolic burden deviates carbon and nitrogen resources from heterologous protein synthesis, and can be expected to result in lower recombinant protein yields. Thus, the use of three individual plasmids was not adequate for the coexpression of three proteins and was not further pursued. To reduce the number of plasmids and auxotrophic markers used, a second approach was devised, where one or three genes were introduced in a single multi copy plasmid. The constitutive promoters PGK1 and TEF1 were used (Figure 1 and Table 1). Genes cloned under these promoters are expected to be constitutively expressed during the whole culture, and expression levels should be independent of the carbon or nitrogen sources used by the cell [20].

**Table 2:** Kinetic and stoichiometric parameters of strains with one (W.T-6) or three (W.TLU-C) plasmids in the same cell

Parameter	Strain	
	
	W.T-6	W.TLU-C
μ_max _(h^-1^)	0.250 ± 0.010	0.159 ± 0.011
T_D _(h)	2.77 ± 0.12	4.33 ± 0.30
Y_x/s _(g DCW/g glucose)	0.133 ± 0.001	0.147 ± 0.002
Culture time (h)	24	36

**Figure 2:**
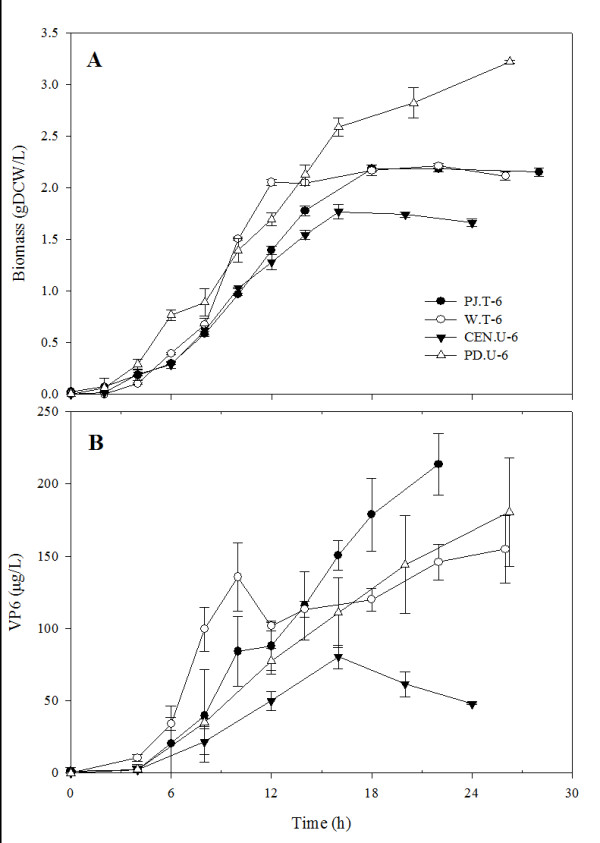
**Batch cultures of *S. cerevisiae *strains producing VP6 in complete synthetic medium**. A) Biomass concentration, measured as dry cell weight and B) VP6 concentration, measured by ELISA (see the analytical methods section). Mean values and standard deviations of three individual cultures are shown.

To identify the best recombinant protein producers, four different yeast strains and two different plasmid variants (YCp and YEp) containing only the VP6 gene were evaluated in batch cultures using CSM without supplementation. Biomass and VP6 kinetics are shown in Figure 2, while kinetic and stoichiometric parameters are listed in Table 3. The strain with the highest growth rate, PD.U-6 started growing before all strains and reached the highest cell concentration. In contrast, growth of the CEN.U-6 strain was delayed and the lowest cell concentration was obtained. The PJ.T-6 strain produced the highest VP6 concentration, which was 2.7 times higher than the VP6 concentration produced by the CEN.U-6 strain. The specific VP6 yield and the VP6 yield on glucose of strain PJ.T-6 were the highest among the strains characterized. In general, VP6 production was growth-associated, but VP6 concentration decreased after glucose consumption in CEN.U-6 cultures (data not shown), possibly as a result of a high protease activity at the end of the culture. A similar behavior has been reported during VLP production in batch cultures after glucose depletion and before ethanol consumption [23]. The lowest specific productivity was observed in strain PD.U-6, while the lowest volumetric productivity was obtained with strain CEN.U-6. Due to the decrease in VP6 concentration observed in cultures of strain CEN.U-6 after glucose depletion and its lower volumetric productivity, strain CEN.U-6 was discarded for the simultaneous production of the three rotavirus proteins and fed batch culture improvements. No effect of the plasmid variant used was observed, even when YEp plasmids have up to 100 copies per cell [24]. Strains PJ.T-6 and PD.U-6 had the highest volumetric productivities in batch cultures. Therefore, their parental strains, PJ69-4a and PD 83B.1d, were used to construct new strains with the multicopy plasmids pWR6 and pWR267 capable of producing VP6 (strains PJ.U-6) and VP2, VP6 and VP7 in the same cell (PD.U-267 and PJ.U-267). These new strains were compared for their heterologous protein production capability in fed-batch culture conditions.

**Table 3:** Kinetic and stoichiometric parameters of batch cultures for VP6 producers

Parameter	Strain			
	
	PJ.T-6	W.T-6	CEN.U-6	PD.U-6
μ_max _(h^-1^)	0.259 ± 0.005	0.276 ± 0.019	0.251 ± 0.035	0.299 ± 0.019
VP6 (μg/L)	214 ± 21	154 ± 23	80 ± 1	181 ± 37
Y_x/s _(g DCW/g glucose)	0.107 ± 0.002	0.105 ± 0.002	0.088 ± 0.003	0.160 ± 0.001
Y_p/x _(μg VP6/g DCW)	99.42 ± 7.95	73.35 ± 9.61	45.47 ± 2.76	56.13 ± 11.46
Y_p/s _(μg VP6/g glucose)	10.68 ± 1.05	7.74 ± 1.16	4.02 ± 0.40	9.03 ± 1.88
Specific productivity (μg VP6/gDCW· h)	4.52 ± 0.36	2.82 ± 0.37	2.84 ± 0.17	2.14 ± 0.44
Volumetric productivity (μg VP6/L·h)	9.70 ± 0.95	5.96 ± 0.89	5.02 ± 0.50	6.88 ± 1.43

### Process strategies for the simultaneous expression of three rotavirus genes

#### Fed batch cultures

**Figure 3:**
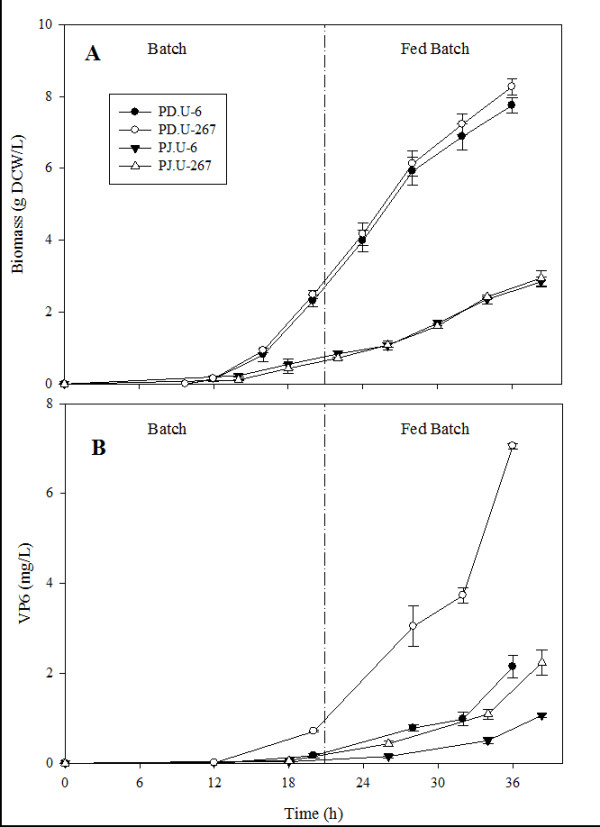
**Fed batch cultures of *S. cerevisiae *strains producing VP6 or VP2, VP6 and VP7**. Complete synthetic medium supplemented with leucine 1.8 mM, glutamate 20 mM and succinate 50 mM was used. A) Biomass kinetics, measured as dry cell weight, and B) VP6 production, measured by ELISA. Mean values and standard deviations of three individual cultures are shown.

**Figure 4:**
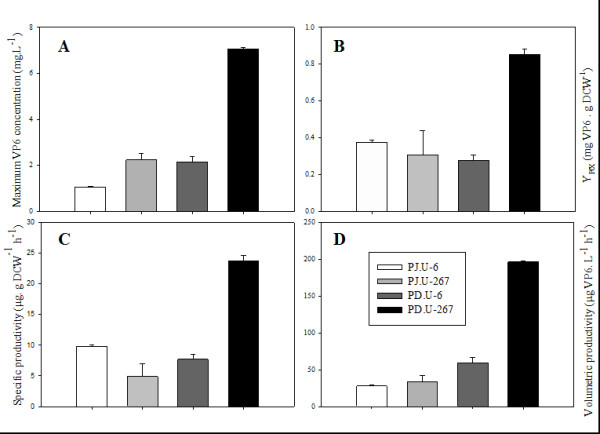
**Product formation and productivities in fed batch cultures of *S. cerevisiae *strains**. A) Maximum VP6 concentration, B) VP6 yields on biomass, C) Specific productivities and D) Volumetric productivities. For details, refer to the Analytical Methods section. Mean values and standard deviations of three individual cultures are shown.

Fed batch cultures of PD.U-6, PD.U-267, PJ.U-6 and PJ.U-267 strains were performed. Biomass concentration and VP6 production were followed during cultures (Figure 3). Yields and productivities are illustrated in Figure 4. As observed in batch cultures, PJ strains grew slower than PD strains before and after the fed-batch stage. Accordingly, maximum biomass concentrations of the PJ.U strains were 3.5 times lower than those of PD.U strains. VP6 concentration before nutrient feeding was less than 1 mg/mL in all strains. VP6 accumulated faster during the feeding stage. Maximum VP6 concentrations obtained ranged from 1 to 7 mg/L in strains PJ.U-6 and PD.U-267, respectively. Interestingly, when VP6 was produced alone, its concentration was three- to two-fold lower than when it was co-expressed with VP2 and VP7 (Figure 4A), regardless of the strain used. Y_P/X _was similar in strains PJ.U-6, PJ.U-267 and PD.U-6, but increased more than three-fold in strain PD.U-267 (Figure 4B), reflecting a more efficient nutrient utilization for heterologous protein production in this strain. Strain PD.U-267 had the highest specific and volumetric productivities (Figures 4C and 4D).

**Figure 5:**
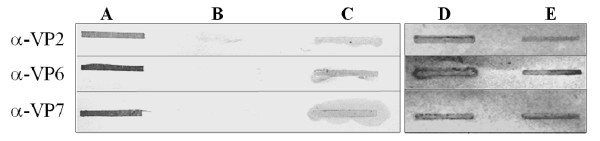
**Immunoblotting of proteins at different purification stages**. A) Inactivated rotavirus strain SA-11 (positive control) B) Extract of a culture of PD.U-C (negative control). C) Yeast extract of the PD.U-267 strain at the end of the fed batch culture. D) Pellet from a sucrose cushion dissolved in TNC buffer. E) A CsCl gradient band. Samples were collected at the end of the fed batch culture and processed as described in the Analytical Methods section. Three monoclonal antibodies were used, 3A8, 255 and IC3 for VP2, VP6 and VP7, respectively. All antibodies bind to conformational epítopes. 200 μL of each sample were loaded onto nitrocellulose membranes.

**Figure 6:**
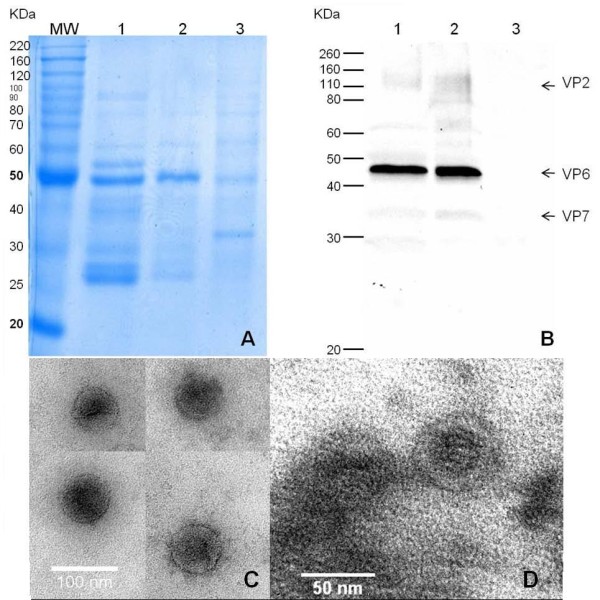
**Analysis of rotavirus-like particles obtained from cesium chloride gradients**. A) and B) SDS-PAGE and Western blot analysis of two opalescent bands isolated from yeast extracts of two different cultures (lanes 1 and 2). Yeast extract of the PD.U-C strain as negative control (lane 3). Each lane was loaded with 5 μg of total protein. C) and D) Transmission electron micrographs of rotavirus-like particles recovered by cesium chloride gradients. Samples were stained with 3% uranyl acetate. Magnification 150,000× and 250,000×, respectively.

Yeast extracts of PD.U-267 were submitted to a sucrose cushion and isopycnic ultracentrifugation to recover tlRLP. The presence of VP2, VP6 and VP7 in different purification stages was detected by immunoblotting using monoclonal antibodies (Figure 5). CsCl bands were analyzed by SDS-PAGE and Western Blot (Figure 6A and 6B), showing the presence of the three rotavirus proteins. Densitometry of SDS-PAGE gels revealed a relative mass ratio of the three recombinant rotavirus proteins VP2:VP6:VP7 of 1:12:4 in samples from gradients band. RLP were observed by TEM in the bands recovered by ultracentrifugation (Figure 6C and 6D). tlRLP had a morphology similar to that previously reported [25]. Measurements of the RLP obtained were performed using the ImageJ software (Wayne Rasband, NIH, USA), and correspond to the expected size (71 ± 5 nm) for RLP. The presence of the three recombinant proteins in the preparations obtained after purification by density gradients and TEM images confirm the successful production of triple-layered RLP in yeast. Low VP7 concentration in the purified bands could be related to a lower quantity of protein produced or inadequate aggregation conditions related to glycosilation patterns and transport phenomena from the ER to assemble into tlRLP.

## Discussion

In this work, strategies for the production of triple-layered VLP in yeast were implemented. Our first attempts to express rotavirus VP6 in batch cultures (data not shown) resulted in concentrations four orders of magnitude lower than those routinely obtained in our laboratory in the insect cell-baculovirus system, ca. 200 mg/L (unpublished results). High recombinant protein concentration is especially important for the production of VLP, as higher concentrations are expected to result in higher assembly efficiency both *in vivo *and *in vitro *methods [1]. Therefore, molecular and process strategies for increasing the concentration of recombinant rotavirus proteins in yeast were designed in this work to allow their assembly into tlRLP. First, the use of three individual plasmids was compared with the use of a single plasmid. The use of three plasmids resulted in an important metabolic burden, as evidenced by a decrease in growth rate in comparison with the strain with a single plasmid. Using a single plasmid for the expression of the three rotavirus genes has several advantages: (a) the copy number is the same for each rotavirus gene, (b) the nucleic acid demand is reduced, and (c) only one auxotrophy needs to be complemented. Several authors have previously reported that the metabolic burden of recombinant protein production in *S. cerevisiae *increase due to plasmid copy number amplification [26-28]. Therefore, the use of a single plasmid expressing the three rotavirus genes was pursued (see Figure 1). Interestingly, no difference in growth rate was observed when the same strain with a single plasmid expressed one or three recombinant proteins (Figure 3A), suggesting that the burden imposed by three plasmids was not due to the simultaneous expression of three recombinant genes, but to the load of plasmid amplification or to the need of complementing three auxothropies.

To select the yeast strain and plasmid to be used for the production of tlRLP, four *Saccharomyces cerevisiae *laboratory strains with different auxotrophic markers and two types of plasmids, YCp (low copy number) and YEp (high copy number) containing only the rotavirus VP6 gene, were evaluated. The CEN.PK. strains have been used for the industrial production of metabolites and some extracellular proteins in laboratory and commercial applications [29]. CEN.PK.113-5D strain has only one auxotrophy for Ura. The W303-1a is a widely used laboratory haploid strain and has 5 auxotrophies (see Table 1). The PD strain was obtained from the W303-1a strain through the repair of four of its auxotrophies, leaving only an auxotrophy for Ura. e PJ69-4a was developed for the two-hybrid system and has four auxotrophies. VP6 production was growth-associated in all strain and plasmid combinations. The best VP6 producers were the PJ.T-6 and the PD.U-6 strains (Table 3). While the PJ.T-6 strain has 5 auxothrophies, the PD.U-6 has only one. All auxotrophies for the PJ.T-6 strain were supplemented, with the exception of Ura. A decrease in VP6 concentration after the onset of the stationary growth phase was observed only in cultures of the CEN.U-6 strain, most likely a result of protease activity. Although in this work no efforts were pursued to identify the proteases involved, other studies have reported a high aspartyl and serine protease activities (PrA and PrB, mainly) in *Saccharomyces *strains expressing a recombinant hepatitis B surface antigen (rHBsAg) [23]. PrA and PrB are located in the vacuole and are released to the extracellular space when cells rupture. Their concentration increases in response to nutritional stress, and results in important protein degradation [30,31]. No effect of the type of plasmid used for the individual expression of VP6 was observed. This behavior could be related to a higher stability of centromeric plasmids in comparison with plasmids based on 2 m sequences, even when the later have higher copy numbers [24].

Selection of the best strain increased recombinant VP6 concentration one order of magnitude. However, VP6 concentration was still three orders of magnitude lower than that obtained in insect cell cultures. Therefore, efforts were aimed at increasing the recombinant protein concentration by using fed-batch cultures, and evaluating two strains that were the best producers in batch cultures. Nutrient feeding increased VP6 concentration 11 times, when VP6 was expressed alone, and 28 times when it was coexpressed with VP2 and VP7. VP6 concentration was three-fold higher when it was co-expressed with VP2 and VP7. This behavior could be associated with a synergistic stabilization of the three structural proteins, protecting them from proteases by their complex structure at the end of the fed batch culture. PJ.U strains grew slower in fed batch cultures and produced less recombinant protein than the PD.U strains. This behavior could be associated to the number of auxotrophies of the PJ.U strain and/or a non-optimized feeding strategy. The maximum VP6 concentration obtained in fed-batch cultures was 144 times higher than the first batch cultures without strain or plasmid selection. The PD.U strains were also the most productive, with a productivity of 196 μg/L h.

The modifications of (a) supplementing limiting metabolites and (b) using a single vector with all three genes increased productivity in batch and fed batch cultures in complete synthetic medium without sacrificing the strong selection pressure that would be lost in complex media. The higher cost associated with defined and supplemented media may be justified on the basis of increased reproducibility, productivity and regulatory requirements for industrial purposes [32].

VLP formation in *Saccharomyces cerevisiae *was evident in transmission electron microscopy images. However, the VP7/VP6 ratio measured by densitometry in SDS-PAGE gels was 59% lower than that present in the triple-layered rotavirus particle (VP7 mass/VP6 mass = 0.83, calculated from the molecular weight of VP7 and VP6). The observed rotavirus protein ratio in CsCl gradients indicates that only 41% of the obtained rotavirus-like particles were triple layered, as a result of an inefficient assembly or expression of VP7. Thus, a challenge that must be overcome for rotavirus VLP expression in yeast is increasing the heterologous protein concentration to promote a more efficient self-assembly into VLP. In the case of recombinant VLP production in *S. cerevisiae*, limitations in accumulation of VLP can result from (a) limitation of raw materials for the synthesis of the different components of VLP, (b) protein degradation processes, (c) inhibition of proper particle formation, (d) physical constraints on volume in the cytosol, among others. These factors may work in synergistic ways to limit productivity, i.e. limitations in particle formation may divert recombinant protein to degradation processes. Volumetric productivities of rotavirus proteins in this study are still one order of magnitude lower than the values reached in insect cells [8]. As the culture media, the production process and the equipment required for recombinant protein production in yeast systems are simpler and less expensive than those used in the insect cell system, we believe that yeast are an attractive option for tlRLP production.

In the present study, the simultaneous production of three proteins aimed to the production of triple-layered VLP is shown for the first time in yeast. Although, tlRLP concentration was very low in comparison with other expression platforms, this study constitutes the basis for economic and easy large-scale production of these complex proteins in an efficient system such as yeast. These developments should be useful for the antigen production in a recombinant veterinary vaccine formulation, but can be extrapolated to human use, when the process conditions and the economic costs associated to downstream processes become competitive.

## Abbreviations

b: Feeding constant (0.19 h^-1^); C^f^_s_: Substrate concentration in the feed (g glucose/L); C_s_: Substrate concentration at time *t *(g glucose/L); CSM: Complete synthetic medium; CTR: Carbon dioxide transfer rate value (mM/h); F: Feeding flow rate (mL/h); IC-BVS: Insect cell/baculovirus system; OD_600_: Optical density measured at 600 nm; PBS: Phosphate buffered solution; PGK1: Phosphoglycerate kinase 1 promoter; PMA1: Plasma membrane ATPase 1 promoter; RBS: Ribosome binding site; rHBsAg: Recombinant hepatitis B surface antigen; RLP: Rotavirus-like particles; TEF1: Transcriptional elongation factor 1 promoter; TEM: Transmission electron microscopy; tlRLP: Triple layered rotavirus-like particles; VLP: Virus-like particles; Vo: Initial volume in batch stage (L); vvm: Gas volume flow per unit of liquid volume per minute (min^-1^); x_0_: Initial biomass concentration at the end of the batch phase (g/L); YCp: Yeast centromeric plasmid; YEp: Yeast episomal plasmid; Y_s/x_: Substrate over biomass ratio (g glucose/g DCW)

## Competing interests

The authors declare that they have no competing interests.

## Authors' contributions

WARL performed the study design and the experimental work, participated in data analysis and manuscript writing. KEJT participated in the design of the expression vectors and the fermentation strategy and in the fermentation experimental work. JN participated in the design of the expression vectors and the fermentation strategy. OTR participated in study design, data analysis and critically revised the manuscript. LAP conceived and coordinated the study, participated in study design, data analysis and manuscript writing. All authors read and approved the final manuscript.
